# Long Short-Term Memory-Based Music Analysis System for Music Therapy

**DOI:** 10.3389/fpsyg.2022.928048

**Published:** 2022-06-14

**Authors:** Ya Li, Xiulai Li, Zheng Lou, Chaofan Chen

**Affiliations:** ^1^College of Music, Hainan Normal University, Haikou, China; ^2^Hainan Hairui Zhong Chuang Technol Co. Ltd., Haikou, China

**Keywords:** music therapy, LSTM, autoencoder, music analysis, psychology, emotion

## Abstract

Music can express people’s thoughts and emotions. Music therapy is to stimulate and hypnotize the human brain by using various forms of music activities, such as listening, singing, playing and rhythm. With the empowerment of artificial intelligence, music therapy technology has made innovative development in the whole process of “diagnosis, treatment and evaluation.” It is necessary to make use of the advantages of artificial intelligence technology to innovate music therapy methods, ensure the accuracy of treatment schemes, and provide more paths for the development of the medical field. This paper proposes an long short-term memory (LSTM)-based generation and classification algorithm for multi-voice music data. A Multi-Voice Music Generation system called MVMG based on the algorithm is developed. MVMG contains two main steps. At first, the music data are modeled to the MDPI and text sequence data by using an autoencoder model, including music features extraction and music clip representation. And then an LSTM-based music generation and classification model is developed for generating and analyzing music in specific treatment scenario. MVMG is evaluated based on the datasets collected by us: the single-melody MIDI files and the Chinese classical music dataset. The experiment shows that the highest accuracy of the autoencoder-based feature extractor can achieve 95.3%. And the average F1-score of LSTM is 95.68%, which is much higher than the DNN-based classification model.

## Introduction

Music consists of four parts: rhythm, melody, harmony, and timbre. It can express people’s thoughts and emotions (Panda et al., 2020). It is nonverbal expression. Music therapy is to stimulate and hypnotize the human brain by using various forms of music activities, such as listening, singing, playing and rhythm (Köhler et al., 2020). It can stimulate the body response by sound to relax the body and mind. From a biological point of view, the human left brain has the function of language, and the right brain has the function of image thinking. For example, music can reduce the emotional and behavioral disorders of the brain by stimulating the human right brain.

There are many advantages of music therapy (Moss, 2019). At first, music can reduce psychological and physical pressure, improve bad emotions, and alleviate physical discomfort caused by emotions. Second music therapy targets a wide range of people. It is an enjoyment therapy without restrictions such as age, gender, and knowledge background. Third, music therapy can relieve the pressure of office workers and is suitable for enterprises to help employees solve psychological and behavioral problems. Fourth, music therapy can be used for mental and physical diseases, shorten the hospitalization cycle of patients, and promote their rehabilitation. For example, music can enhance people’s memory and attention, relax people’s body, and mind, and reduce depression and anxiety; Patients can follow the rhythm of music for a long time. Patients with headache can use music therapy for pain management and reduce the use of painkillers. Fifth, music therapy can stimulate the brain, activate brain cells, greatly help brain activities, and even prevent aging, especially for brain dysfunction, Alzheimer’s disease, Parkinson’s disease, post-traumatic consciousness disorder, etc. After music therapy, such patients can improve their cognitive and motor functions. Sixth, music helps to improve people’s sleep quality, increase nerve conduction rate, and relax people’s body and heart.

With the empowerment of artificial intelligence, music therapy technology has made innovative development in the whole process of “diagnosis, treatment and evaluation” (Ramirez et al., 2018). Although the effect of traditional music therapy has been generally recognized and accepted, its existing technical means still have some defects, such as inaccurate targeting (e.g., lack of pathological pertinence, ignoring individual differences), time-consuming and laborious (e.g., high labor cost, limited site), low Professionalism (e.g., unsystematic efficacy evaluation indicators), and privacy disclosure (Shi and Zhang, 2020). The rapid development of natural language processing, machine vision, speech recognition and other fields provides ideas for the whole process innovation of “diagnosis treatment evaluation” of music therapy (Qu and Xiong, 2012).

For clinical treatment, music prescription plays an important role (Liu et al., 2021). In the process of music prescription optimization, it mainly includes two aspects. First, the functional music is reasonably classified, and the content is sorted out through the system. It is necessary to establish a music therapy library in the initial stage, which can complement classical music and popular music to ensure the comprehensiveness and scientificity of the music database. Secondly, after understanding the basic framework of music genre, rhythm, musical instrument, emotion and beat, we can choose the corresponding music in the face of different mental diseases, to achieve the ultimate purpose of treatment. For example, in the process of body relaxation treatment, we can choose soothing music, focus on the description of the treatment scene, control the patient’s emotion, and achieve the ideal treatment goal. It is necessary to change the technical means of music therapy in time. For example, we can use the speech synthesis technology to guide treatment. We can also use natural language to complete music lyrics in time, enrich music scores and optimize the performance process. The computer simulation system is used to assist patients in singing songs, to achieve the purpose of respiratory training. From the above introduction, we need to take advantage of the artificial intelligence technology to innovate music therapy methods, enhance the accuracy of treatment schemes, and improve the development of the medical field (Lee et al., 2021).

The current research mainly focuses on the generation and classification of melody (Verhoef and Ravignani, 2021). There is not much research on Multi-Voice Music Generation (MVMG) and classification by considering the collocation of melody and chords. This paper proposes an long short-term memory (LSTM)-based generation and classification algorithm for multi-voice music data. A Multi-Voice Music Generation system called MVMG based on the algorithm is developed. MVMG contains two main steps. At first, the music data are modeled as the MDPI and text sequence data by using an autoencoder model, including music features extractions and music clip representation. And then an LSTM-based music generation and classification model is developed for generating and analyzing music in specific treatment scenario. MVMG is evaluated based on three datasets collected by us: the single-melody MIDI files, the single-melody MIDI files composed by 5400 composers, and the Chinese classical music (CCM). The experiment shows that the highest accuracy of the autoencoder-based feature extractor can achieve 95.3%. And the highest average F1-score of LSTM is 95.68%, which is much higher than the DNN-based classification model. Our main contribution is as follows:

(1) Develop an LSTM-based generation and classification algorithm for generating and analyzing music in specific treatment scenario.
(2) Develop an auto-encoder model to extract features of the music data.
(3) Conduct comprehensive experiments to evaluate the proposed MVMG system based on different types of music datasets.

The structure of the rest of the paper is as follows: Section “Related Work” conducts literature review on the technologies of AI-based music generation and classification. Section “Music Data Modeling and LSTM-Based Music Data Sequence Classification” presents the detailed information of MVMG, including music data modeling and LSTM-based music data sequence classification. Section “Dataset Description and Experiment Analysis” shows the training and testing process of MVMG. It mainly compares the MVMG with DNN-based music generation models. Section “Conclusion” summarizes this work.

## Related Work

Many research works have been conducted in AI-based musical analysis. De Mantaras and Arcos (2002) discussed three musical analysis systems based on machine learning techniques. This research work contributes to the SAXEX analysis. A computer-human interaction platform is designed to produce the music expression like that of human. Frid et al. (2020) proposed a system based on artificial intelligence technologies to rephrase a music. The system has a user-friendly interface. Users can efficiently interact with the system. The video makers can benefit from this system to create health music. Their experiment shows that their system can help communications between people and music analysis systems. Louie et al. (2020) developed an intelligent system to help system users to generate music. The system user can generate a voice sound with emotions such as happy, unhappy, and surprised. They took many experiments to evaluation the proposed system. The results show that the usage experience of the system users can be efficiently improved. In addition, a significant issue of composition frustration of music can be solved by the proposed system. Tan and Li (2021) discussed deep learning-based algorithms for music generation. They presented how to use different deep learning models to composite music and melodies. Other styles of music are also analyzed by deep learning models. They summarized that deep learning technologies can make the music composition more efficient.

Pandian (2019) developed an AI system to evaluate the sleeping pattern based on the therapy of music to improve the quality of sleeping. The data is collected and transferred to the AI platform. The platform can be automatically controlled by analyzing the collected data, which improves the quality of the person’s sleeping. Kumar et al. (2020) proposed an architecture to improve the efficiency of the music generation system. They surveyed different types of algorithms for composing and generating music. They discussed the advantages, disadvantages, and challenges in AI-enabled music generation area. They then proposed an AI-enabled system to compose music, which improves the development of intelligent music generation techniques. Lupker and Turkel (2021) analyzed the pros and cons of the intelligent music generation algorithms based on big musical data. Music theory can be used to reveal the efficiency of the melody prediction algorithm. The performance can be further enhanced. Various intelligent algorithms can be learned from the music data to understand the music.

## Music Data Modeling and Long Short-Term Memory-Based Music Data Sequence Classification

### Multi-Piece Music Data Modeling

Music data is a time series data with a very complex structure. To use the algorithm to generate music data, we must first understand the structural characteristics of music data and the expression of music information. This paper analyzes the characteristics of multi-part music based on the expression methods commonly used in modern music, e.g., MIDI format and Piano Roll format, and establishes a music data model.

MIDI (Music Digital Interface) is a technical standard that describes the connection protocol between computer digital interfaces and various musical instruments. Compared with text formats, MIDI carries a larger amount of information. Modern music is basically synthesized using MIDI. Its basic idea is to express the note information of different pitch and length combinations as events, and also carry information such as the volume and start time of the note, quantify the basic characteristics of music, and put the music in the performance. Each instrument of MIDI is represented as a channel, which records the way the notes of each instrument are played. There are two basic note events in MIDI: note on and note off event. Note on event indicates that a note with a certain pitch and duration starts to be played by a specific instrument in a certain channel. For example, < Note on, 0, 60, 40 > indicates that it starts to be played after 40 units of time in channel 1 middle C sound. Note off event means that a specific instrument in a channel stops playing a note with a certain pitch and length, such as < Note off, 0, 60, 30 > means that in channel 1, stop playing middle C after 30 units of time sound.

The Piano Roll representation method is inspired by the player piano, and its essence is a continuous paper roll that records information by perforating it. Each punch represents a note control message used to trigger a given note. The length of the perforations corresponds to the duration of the note. The positioning of the perforations corresponds to their spacing. A column of perforations represents a key in the piano.

According to the introduction of the MIDI format and Piano Roll format above, it is relatively easy to design a multi-part music expression. That is, a matrix composed of 0 and 1 is used to represent the state information of each note in the multi-part music, where the rows represent the 88 keys in the piano and the columns represent the time series. 1 means the key is down, 0 means the key is up. But this method has a drawback, that is, when there is a series of 1s, it is impossible to tell whether it is played multiple times or a long-lasting note. Therefore, the original piano key dimension of 88 can be expanded to 176 by combining the idea of Note on and note off events in MIDI format. Use the 1 in the first 88 dimensions to indicate that the key is pressed, which is the Note on event, and use the 1 in the last 88 dimensions to indicate that the key is up, which is the Note off event.

This method cannot only effectively distinguish between long notes and multiple playing, but also facilitate the mutual conversion of MIDI music data and Piano Roll matrix. However, through practice, it is found that although this multi-part music modeling method can use the piano key state vector at the same time to represent the playing state of melody and chord, it cannot represent the matching information of melody and chord in the time dimension. That is, the relationship between notes is expressed independently.

### The Representation of Music Features

Using the original Piano Roll format for the expression of multi-voice music data cannot represent the higher-dimensional combination characteristics of melody and chords. Therefore, we use the method of compressing note states at the same time step to extract the relationship between each note state as an implicit feature of music. We use it as a data model for multi-voice music.

An autoencoder is a neural network with one hidden layer. Its special structure is that the number of neurons in the input layer and the output layer of the network are the same. And the number of nodes in the hidden layer is smaller than that in the input and output layers. When training an autoencoder, traditional neural network training methods can be used. The only difference is that the input data for training the autoencoder is the target data to be output. Therefore, the autoencoder learns a representation function of the data. Because the hidden layer has fewer nodes than the input layer, the encoder part must compress the information lossy, while the decoder part needs to reconstruct the original information as much as possible according to the compressed feature information. This forces the autoencoder to discover important distinguishing points in the data, so autoencoders are usually used to extract higher-dimensional features in the data.

With the advent of deep learning, stacked autoencoders have become more widely used. The stacked autoencoder is composed of multiple autoencoders nested, and the number of neurons in the hidden layer decreases sequentially. In this structure, the encoder continuously compresses the data to extract higher-level features. Therefore, this structure similar to the deep neural network is one of the commonly used feature extraction methods.

**FIGURE 1:**
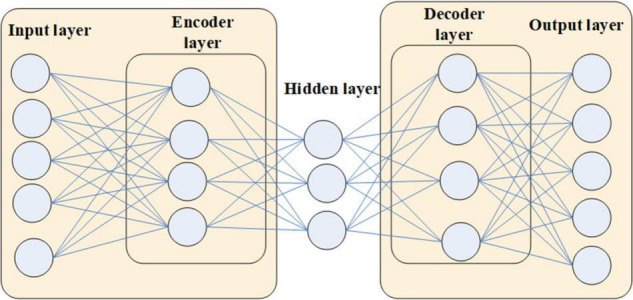
Structure of the autoencoder.

We extract 40 pieces of music data in MIDI format to train the stacked autoencoder. Through the conversion relationship between the Piano Roll format and MIDI data, the Piano Roll matrix of 40 pieces of music is extracted. In the experiment, it is found that due to the similar style of the extracted music data, the pitches of the music are all between 20 and 98. Only the middle 59 keys are used out of the 88 keys of the piano. To reduce the computational complexity, we reduce the original 160-dimensional Piano Roll matrix to 150-dimensional and builds a stacked autoencoder based on the music data in this format, as shown in Figure 1.

The neuron nodes of the input layer and output layer of the stacked autoencoder are both the piano key dimension 150 of the Piano Roll. For the hidden layer, it is necessary to design several nodes smaller than the number of nodes in the input and output layers and adjust them one by one through subsequent training practice.

According to the previous analysis, the stacked autoencoder to be built in this paper is to perform feature extraction on each 150-dimensional 0 and 1 vector of the Piano Roll matrix. After going through the decoder, it should produce the same output as the input vector. Therefore, we adopt the cost function of the training network as the mean square error cost function (Eq. 1).


(1) $$\text{c}=\frac{1}{2\,n}\,\sum_{j=1}^{n}{\left(\gamma_{j}-y_{j}\right)}^{2}$$

where γ is the output result of the network; *y* is the target value, i.e., the input vector; *n* is the number of samples for each training.

### Music Clips Representation

The information in the music clips is converted into natural language sequences in chronological order. This information includes: “n_[pitch]”: The pitch is an integer between 0 and 256 (including 0 and 256). i.e., pitch=1,⋯,256. “q[temporal value]_[dotted value]”: The temporal value is two whole notes, whole notes (whole), half notes (half), quarter notes (quarter), eighth notes (eighth), sixteenth notes (14th), and thirty-second notes (28nd). The number of dots is 0, …, 3. “v[vel]”: The velocity is a multiple of 3 between 3 and 256. i.e., velocity=3,6,⋯,256. “t[sp]”: time of speed. The speed is a multiple of 3 between 18 and 150. i.e., sp=18,2,⋯,150. “.”: End of time step. Each time step is the same length as a sixteenth note. “\n”: The end of the music segment.

### Long Short-Term Memory-Based Music Classification Model Based on Implicit Music Features

RNN is an extension of the fully connected neural network. Based on the fully connected neural network, the nodes of the hidden layer are connected. That is, the input of each hidden layer node is no longer only the output of the previous layer node, but also the hidden state of the node at the last time. Because of its special structure, RNN is different from fully connected neural network in backward propagation training. In the process of training RNN, a special learning method, back propagation through time algorithm is used. The input and output of RNN can be understood as a sequence. Therefore, in the backward propagation training, the nodes can be expanded according to the time series to obtain a structure similar to the fully connected neural network. On the expanded structure, the truncated sequence is used for backward propagation training. In addition to the hidden state value, LSTM also stores the cell state C value inside the node, which is used to represent the long-term dependence and update of information and adds 3 gate structures to control the update size of the node state. The specific calculation formula is as follows:


(2) $$y_{t}=\sigma \,\left(W_{f}\,\left[x_{t},g_{t-1}\right]\right)$$

(3) $$i_{t}=\sigma \,\left(W_{i}\,\left[x_{t},g_{t-1}\right]\right)$$

(4) $$\tilde{p_{t}}=t\,a\,n\,h\,\left(W_{c}\,\left[x_{t},g_{t-1}\right]\right)$$

(5) $$p_{t}=y_{t}\cdot p_{t-1}+i_{t}\cdot \tilde{p_{t}}$$

(6) $$o_{t}=t\,a\,n\,h\,\left(W_{o}\,\left[x_{t},g_{t-1}\right]\right)$$

(7) $$g_{t}=o_{t}\cdot t\,a\,n\,h\,\left(C_{t}\right)$$

The results of the three gates are all obtained from the input *x_t_* and the hidden state *g*_*t–1*_ at the previous moment. The first gate is the forget gate *y*, which decides how much information to discard from the past value of *p* at the current moment. The second gate is the input gate *i*, which determines how much information to store in the *p* value at the current moment. New information to be added is indicated by the symbol $\tilde{p_{t}}$. According to the control coefficients of the forget gate and the input gate, and the new information to be added to the cell, update the current cell state to get *p_t_*. The third gate is the output gate *o*, which updates the current hidden state *g_t_* according to the cell state *p_t_* at the current moment and the output gating information *o_t_*. Equations 2–7 are the complete forward propagation process of LSTM, where *W_y_*, *W_i_*, *W_o_*, and *W_g_* are the weights that need to be trained.

## Dataset Description and Experiment Analysis

### Dataset Information

The training data set consists of three parts of the development data set: (1) the single-melody MIDI files in the Conference on Sound and Music Technology (CSMT) competition (Zhang et al., 2022), (2) the single-melody MIDI files composed by 5400 composers, and (3) the CCM. The CSMT competition development data set only contains music generated by artificial intelligence algorithms, including 6000 single-melody MIDI files, the tempo is between 68 and 118 bpm, each melody is 8 bars in length, and does not contain the complete phrase structure. The melody is generated by the algorithm after training several different music generation models from two databases with completely different musical styles. Due to the lack of the composer’s composition data set, the team obtained 5400 single-melody MIDI files through the open-source website search, manual scoring, audio file conversion, and multi-melody stripping of the main melody. The structure of these files is more consistent with the official development data set. The evaluation data set is the official 2000 MIDI files, all settings are the same as the training set.

The dataset of CCM consists of 71 CCM songs, which are transcribed from the piano scores. The piano scores we collected are from the recently published CCM music collections in the form of notation. The content in CMM is the numbered melody in the piano score and the harmonic marks in it. In the musical notation, the pitch is written in the way of the first key roll call. That is, the first order note of the current mode is C or 1, and the absolute pitch of the middle pitch of the piano score must be jointly determined by the tuning pitch. A CCM piece usually contains multiple paragraphs. In the CCM data set, each paragraph is a separate file. The dataset contains a total of 380 segments and 8900 subsections of data.

We annotated the music scores in the CCM dataset with meta-information, including the name of the score, the tuning, the source of the score, the source of the music, the performer, and the scorer or notator. Although the CMM pieces vary in length, most of the CCM scores we collected are shorter than 150 bars. At the same time, the scores we collect often contain section marks and are divided into sections. Most of these paragraphs are relatively short, less than 30 bars in length. There are a few paragraphs in the histogram that are very long, because there are no paragraph markers in these scores and the entire piece is treated as one paragraph. In CCM, 10% of the notes are chords, and 90% of the chords contain two pitches. It is worth noting that the overall chord ratio in the CCM music may be slightly higher than this dataset because we avoid some difficult-to-enter scores containing more chords.

### Performance Evaluation of the Autoencoder-Based Feature Extraction Model

The autoencoder model is trained using the music clips in the training dataset as samples. To diversify the training samples, a series of transformations are performed on these music pieces, including time transformation (speed up, slow down), pitch transformation (each note is raised or lowered by a major third). These sequences are stitched together and randomly divided into training and test sets in an 8:2 ratio. The training set is then equally divided into four subsets for subsequent processing. Each training subset includes approximately 17,600 music samples, and the test set includes approximately 5400 samples. The autoencoder model is trained sequentially using the training subset. Both g_t_ and p_t_ are initialized to 0 before each training. After 5 rounds of training, the Adam method is used for optimization, and the average cross-entropy loss on the test set is 0.72.

To test the feature extraction effect of the self-encoder in music data other than the training data set, another three MIDI music with the same style are used to test the network. Encode and decode the note state data of music and compare the output value of the network with the real value. The accuracy is shown in Table 1.

**TABLE 1:** Accuracy of the stacked autoencoder.

Sample	Accuracy (%)
Music1	91.8
Music2	95.3
Music3	92.9
	

It can be seen from Table 1 that the stack autoencoder has a high accuracy of decoding after encoding in this style of music data, which means that the encoder has extracted better implicit music features. By using the encoder part of the stacked autoencoder to perform implicit feature extraction on the Piano Roll matrix of the music data, the multi-voice music data model is well-trained.

### Performance Evaluation of Long Short-Term Memory-Based Classification Model

The LSTM model trained in the previous step can be used as a special encoder. To balance and diversify the number of samples, samples of AI-created and composer compositions in the MIDI database are time-shifted (speed up, slow down) and pitch-shifted (all notes up, down a major third). Input these music samples into the LSTM model, take the final cell state (4096-dimensional vector) of the LSTM layer in the model as the encoding result, and use logistic regression for classification. The performance of the model was evaluated using 10-fold cross validation and the results are shown in Table 2.

**TABLE 2:** Results for 10-fold cross validation.

Rounds	Accuracy (%)
1	97.4007
2	97.4226
3	97.3787
4	97.4592
5	97.0813
6	97.6789
7	97.4592
8	97.4971
9	97.0474
10	97.7659

The confusion matrix and classification report that works best on the test set are shown in Tables 3, 4.

**TABLE 3:** Confusion matrix of the highest accuracy test set.

ActualForecast	DNN	Composer
MVMG	700	0
DNN	2	623
		

**TABLE 4:** Classification report of the highest accuracy test set.

	Precision	Recall	F1 score
MVMG	0.9237	0.9981	0.9568
DNN	0.8882	0.9237	0.9045

As can be seen from the above table, the f1 of MVMG is 95.68%. It shows that LSTM is an effective classifier, which can include the approximate information of a music sample through a 4096-dimensional vector. LSTM has excellent performance on artificial intelligence and composer composition classification tasks. The LSTM model is trained by supervised learning, which does not require many labeled samples during training. It is thus convenient for use when the cost of label acquisition is high. It can effectively extract the features of symbolic music and has a high accuracy in classifying the music creation period. Since the original purpose of the LSTM model is to improve the accuracy of the characters at the next moment in the prediction sequence, it can also be used to complete music generation task.

## Conclusion

This study focuses on the generation and classification of melody. There is not much research on MVMG and classification by considering the collocation of melody and chords. We proposed an LSTM-based generation and classification algorithm for multi-voice music data. A Multi-Voice Music Generation system called MVMG based on the algorithm is developed. MVMG contains two main steps. At first, the music data are modeled as the MDPI and text sequence data by using an autoencoder model, including music features extractions and music clip representation. And then an LSTM-based music generation and classification model is developed for generating and analyzing music in specific treatment scenario. MVMG is evaluated based on three datasets collected by us: the single-melody MIDI files, the single-melody MIDI files composed by 5400 composers, and the CCM. The experiment shows that the highest accuracy of the autoencoder-based feature extractor can achieve 95.3%. And the highest average F1-score of LSTM is 95.68%, which is much higher than the DNN-based classification model. In the future, we are going to develop a more advanced feature extraction model by combining convolutional layers with auto-encoders. More accurate feature extraction can improve the classification accuracy.

## Data Availability Statement

The original contributions presented in this study are included in the article/supplementary material, further inquiries can be directed to the corresponding author.

## Author Contributions

YL performed the experiment and wrote the manuscript. XL discussed the idea with YL and improved the idea. ZL collected the data. CC proof-read the manuscript. All authors contributed to the article and approved the submitted version.

## Conflict of Interest

XL and CC were employed by Hainan Hairui Zhong Chuang Technol Co. Ltd. The remaining authors declare that the research was conducted in the absence of any commercial or financial relationships that could be construed as a potential conflict of interest.

## Publisher’s Note

All claims expressed in this article are solely those of the authors and do not necessarily represent those of their affiliated organizations, or those of the publisher, the editors and the reviewers. Any product that may be evaluated in this article, or claim that may be made by its manufacturer, is not guaranteed or endorsed by the publisher.
